# PREscribing preoperative weight loss prior to major non-bariatric abdominal surgery for patients with Elevated weight: Patient and Provider Survey Protocols (PREPARE surveys)

**DOI:** 10.1371/journal.pone.0302482

**Published:** 2024-04-30

**Authors:** Tyler McKechnie, Maisa Saddik, Aristithes Doumouras, Cagla Eskicioglu, Sameer Parpia, Mohit Bhandari

**Affiliations:** 1 Department of Surgery, Division of General Surgery, McMaster University, Hamilton, Ontario, Canada; 2 Department of Health Research Methods, Evidence and Impact, McMaster University, Hamilton, Ontario, Canada; 3 Department of Surgery, Division of General Surgery, St. Joseph Healthcare, Hamilton, Ontario, Canada; 4 Department of Oncology, McMaster University, Hamilton, Ontario, Canada; 5 Department of Surgery, Division of Orthopedic Surgery, McMaster University, Hamilton, Ontario, Canada; IRCCS: IRCCS Ospedale San Raffaele, ITALY

## Abstract

**Background:**

Preoperative very low energy diet (VLED) interventions are used routinely in patients undergoing bariatric surgery, a surgical subspecialty that deals almost exclusively with patients with obesity. Yet, their use and study has been limited in non-bariatric abdominal surgery. To investigate the use of VLEDs in non-bariatric surgery, we plan on conducting a randomized controlled trial (RCT). Prior to proceeding, however, we have designed two surveys as important pre-emptive studies aimed at elucidating patient and provider perspectives regarding these interventions.

**Methods:**

The patient survey is a cross-sectional, single-center survey aimed at assessing the safety, adherence, barriers to adherence, and willingness to participate in preoperative optimization protocols with VLEDs prior to undergoing elective non-bariatric intra-abdominal surgery (S1 File). The population of interest is all adult patients with obesity undergoing elective non-bariatric intra-abdominal surgery at St. Joseph’s Healthcare Hamilton who were prescribed a course of preoperative VLED. The primary outcomes will be safety and adherence. The target sample size is 35 survey responses. The provider survey is a cross-sectional national survey of practicing surgeons in Canada who perform major non-bariatric abdominal surgery aimed assessing the willingness and ability to prescribe preoperative weight loss interventions amongst practicing Canadian surgeons who perform major non-bariatric abdominal surgery (S2 File). The population of interest is independent practicing surgeons in Canada who perform major non-bariatric abdominal surgery. The primary outcome will be willingness to prescribe preoperative VLED to patients with obesity undergoing major non-bariatric abdominal surgery for both benign and malignant indications. The target sample size is 61 survey responses. Descriptive statistics will be used to characterize the sample populations. To determine variables associated with primary outcomes in the surveys, regression analyses will be performed.

**Discussion:**

These survey data will ultimately inform the design of an RCT evaluating the efficacy of preoperative VLEDs for patients with obesity undergoing major abdominal surgery.

## Introduction

Obesity is a global epidemic [1]. By 2030, it is projected that half of the United States population will have a body mass index (BMI) over 30kg/m^2^ [2]. While there are many associated downsides, such as increased risk of cardiovascular disease, diabetes, and cancer, in surgery these patients present significant perioperative challenges [3–5]. Intraoperatively, increased BMIs are associated with increased operative time and estimated blood loss [6,7]. Postoperatively, patients with obesity are at greater risk of infectious complications, wound complications, cardiovascular complications, and more [8,9]. These challenges are particularly heightened in patients undergoing major abdominal surgery [10]. In an attempt to pre-empt these perioperative challenges, a group of surgeons at our institution have begun prescribing preoperative very low energy diets (VLEDs) with liquid formulation (e.g., Optifast® 900). These interventions are used routinely in patients undergoing bariatric surgery, a surgical subspecialty that deals almost exclusively with patients with obesity [11]. Yet, their use and study has been limited in non-bariatric abdominal surgery.

We conducted a systematic review aimed at identifying studies that evaluated VLEDs in non-bariatric surgery [12]. Thirteen studies were found, nine of which evaluated patients with obesity undergoing abdominal surgery. While the evidence was heterogenous, the available data suggests preoperative VLEDs are safe, well tolerated, and result in significant preoperative weight loss for these patients. None of these included studies thoroughly evaluated the patient or provider perspectives with sound survey methodology. As such, prior to proceeding with a randomized controlled trial (RCT) aimed at assessing the efficacy of VLEDs at reducing operative difficulty and improving postoperative outcomes, we have designed two surveys aimed at elucidating patient and provider perspectives regarding these interventions. We believe the patient survey is necessary given the potential barrier that patient adherence to these interventions may pose to the successful conduct of an RCT in this field. By performing the first study focused solely on the patient experience with preoperative VLEDs prior to major non-bariatric abdominal surgery, we will be able to elucidate important granular detail pertaining to patient adherence to this intervention. Another essential component for the successful conduct of an RCT is clinical equipoise. While our previous systematic review has suggested the need for a large trial to resolve uncertainty pertaining to the use of VLEDs prior to major non-bariatric surgery, the current provider survey will aid in establishing clinical equipoise and willingness to prescribe preoperative VLEDs [12].

The first survey in this protocol is a cross-sectional, single-center survey that was designed to assess patient perspectives on safety, adherence, barriers to adherence, and willingness to participate in preoperative optimization protocols with VLEDs prior to undergoing elective non-bariatric intra-abdominal surgery. Local surgeons have been routinely prescribing preoperative VLEDs for patients with obesity undergoing non-bariatric surgery since 2018, thus this will serve as the population of interest for the patient survey. The second survey is cross-sectional national survey of practicing surgeons in Canada who perform major non-bariatric abdominal surgery. The objective of the second survey is to assess current practice patterns in independent practicing surgeons across Canada in terms of preoperative weight loss interventions for patients with obesity undergoing non-bariatric abdominal surgery. We hypothesize that the patient survey will find patient-reported adherence of less than 100% with preoperative VLEDs and there will be common barriers to adherence reported (e.g., hunger, cost, lack of obvious benefit at onset of intervention). Nonetheless, we anticipate that VLED-associated adverse events will be relatively low and in keeping with previously published data (~32.5%) [12]. With regards to the provider survey, we anticipate a low proportion of practicing surgeons will be routinely prescribing preoperative VLEDs but that willingness to prescribe will be high (i.e., median response of 4 on a 5-point Likert scale). These findings will inform a subsequent RCT evaluating preoperative VLEDs for patients with obesity undergoing non-bariatric surgery which ultimately can inform a preoperative optimization pathway for these patients with the aim of improving outcomes and decreasing associated healthcare burden.

## Materials and methods

### Study objectives

#### Patient survey

The principal research question is: Are preoperative VLEDs with Optifast® 900 (intervention) safe, well adhered to, and well tolerated (outcomes) in adult patients with obesity (i.e., older than 18 years of age and BMI greater than 30 kg/m^2^) undergoing elective non-bariatric intra-abdominal surgery (population)?

The specific aims of this patient survey will be: (1) Determine patient-reported adverse effects of participating in preoperative protocols that include preoperative VLEDs with liquid formulation ***(Safety)***; (2) Determine patient-reported adherence with preoperative VLEDs with liquid formulation prior to elective non-bariatric abdominal surgery ***(Adherence)***; (3) Determine barriers to adherence with preoperative VLEDs with liquid formulation ***(Adherence)***; (4) Determine thresholds for outcome differences at which patients would be willing to adhere with preoperative VLEDs with liquid formulation ***(Adherence)***; (5) Determine the amount of patient reported preoperative weight loss with the preoperative use of VLEDs ***(Efficacy)***; (6) Determine the impact of preoperative VLEDs with liquid formulation on health-related QoL in adult patients with obesity undergoing non-bariatric abdominal surgery ***(Quality of Life [QoL])***; and (7) Inform the design of a multi-center RCT assessing the use of preoperative VLED ***(Feasibility)***.

#### Provider survey

The principal research question is: Are Canadian surgeons who perform elective non-bariatric intra-abdominal surgery (population) willing to prescribe preoperative weight loss by way of VLEDs (outcome) for patients with obesity undergoing non-bariatric abdominal surgery according to self-reported survey data (intervention)?

The specific aims of this provider survey will be: (1) Determine willingness to prescribe preoperative VLED to patients with both benign and malignant diseases undergoing non-bariatric abdominal surgery ***(Feasibility)***; (2) Determine the frequency at which preoperative weight loss interventions are prescribed for patients with obesity undergoing non-bariatric abdominal surgery ***(Feasibility)***; (3) Determine the different types of preoperative weight loss interventions that are being used in Canada ***(Feasibility)***; and (4) Inform the design of a multi-center RCT assessing the use of preoperative VLED ***(Feasibility)***.

### Study design

#### Patient survey

This is a cross-sectional, single-center survey.

#### Provider survey

This is a cross-sectional national survey.

### Survey sample

#### Patient survey

The population of interest is all adult (i.e., 18 years of age or older) patients with obesity (i.e., BMI greater than 30kg/m^2^) undergoing elective non-bariatric intra-abdominal surgery at St. Joseph’s Healthcare Hamilton who were prescribed a course of preoperative VLED. If the first postoperative visit is within two months of their operative date, patients will be considered for inclusion. Patients who underwent bariatric surgery and surgeries other than intra-abdominal surgery will be excluded. Patients who are unable to either read, write, or speak in English or have any comorbidities, impairments, or disabilities prohibiting them from completing the survey will be excluded.

#### Provider survey

The population of interest is all independent practicing surgeons in Canada who perform major non-bariatric abdominal surgery. Surgical sub-specialties that will be eligible for inclusion will be general surgery, colorectal surgery, hepatobiliary surgery, surgical oncology, thoracic surgery, vascular surgery, urology, and gynecology. Surgeons who perform bariatric surgery exclusively will be excluded. Surgeons who are unable to respond to the survey in English or French will be excluded. Incomplete survey responses will be excluded.

### Sampling technique

#### Patient survey

Our sampling strategy will be non-probability convenience sampling in which we will approach consecutive patients presenting to our surgical clinics at St. Joseph’s Healthcare Hamilton for their first postoperative visit. Non-probability sampling refers to a non-random sample selection, of which convenience sampling is one particular sub-type. Surgeons will introduce the research study to patients presenting to their initial postoperative clinic visit. If the patient is agreeable to learning more about the study, the research team will be notified. They will confirm eligibility and then, should the patient be eligible, proceed with the informed consent process. Immediately following this, the researcher will implement the survey. The survey will be kept as a hard-copy paper survey as well as converted to an electronic form on RedCap®. For patients presenting to their first postoperative visit (i.e., within two months of their operative date) in person, we will distribute a hard-copy paper survey to be completed in clinic. For patients with a virtual postoperative visit, we will obtain their email address and distribute an electronic cope of the survey to be completed on RedCap® at their earliest convenience.

#### Provider survey

The sampling strategy for this survey will also be non-probability convenience sampling. To distribute the survey as broadly as possible, we will distribute the electronic survey via email through provincial and national surgery associations (e.g., Canadian Association of General Surgeons, Canadian Urology Association, Society of Obstetricians and Gynecologists, Ontario Association of General Surgeons, Association Québécoise de Chirurgie, etc.) listservs. If the response rate remains low (i.e., sample size goal not reached within 3 months) after distribution through these channels, we will contact individual departments of surgery at each academic institution across Canada (i.e., University of British Columbia, University of Calgary, University of Alberta, University of Saskatchewan, University of Manitoba, Northern Ontario School of Medicine, University of Western Ontario, McMaster University, University of Toronto, Queen’s University, University of Ottawa, McGill University, University of Sherbrooke, University of Montreal, Laval University, Dalhousie University, Memorial University) as well as the corresponding divisions of General Surgery to distribute to their members.

#### Survey design

The surveys will be designed according to the Canadian Medical Association Journal *Guide for the Design and Conduct of Self-Administration Surveys for Clinicians* [13]. A thorough literature review informed survey questions. Content experts (one colorectal surgeon, one general surgeon, and two bariatric surgeons) collaborated to develop survey questions. Prior to production of the final version of the surveys, methodology was critically appraised by two PhD biostatisticians. The patient survey was piloted by five bariatric surgery patients who had completed a preoperative VLED prior to their operation. The provider survey was piloted by five local practicing surgeons prior to dissemination to the sample population. Previous survey methodology research has demonstrated the importance of piloting surveys in an attempt to reduce bias and improve accuracy [14]. Pilot responses will not be included in the final analyses. The provider survey was translated to French by a member of the study team who is a practicing General Surgeon in Montreal and is bilingual.

### Data collection

#### Patient survey

The patient survey will collect data as per the following survey sections: (1) Demographic information (e.g., age, sex, location of residence); (2) Treatment information (e.g., date of surgery, type of disease, type of surgery, operative approach); (3) Prescribed preoperative weight loss information (e.g., duration of VLED, adherence with VLED, barriers to adherence, VLED-associated adverse events); and (4) Health-related QoL (i.e., according to the Short-Form 12).

#### Provider survey

The provider survey will collect data as per the following survey sections: (1) Demographic information (e.g., age, sex, number of years in practice, location of practice, type of practice); (2) Institution information (e.g., number of years in practice, location of practice, type of practice, availability of bariatric surgery, availability of dietician, availability of non-surgical preoperative clinics); (3) Preoperative weight loss prescribing practices, willingness, and knowledge (e.g., is prescribing preoperative weight loss a routine part of practice, what types of preoperative weight loss interventions are prescribed, what BMI cut-off is utilized); (4) Questions specific to patients with oncologic disease (e.g., apprehension with regards to prescribing to patients with oncologic disease, willingness to prescribing to patients with oncologic disease).

### Outcome measures

#### Patient survey

The primary outcomes will be safety and adherence. Safety will be assessed through VLED-associated adverse events. The adverse events will be recorded as dichotomous outcomes and described as either minor or serious, in a similar fashion to the OPTIWIN Study; the largest medical weight loss RCT evaluating Optifast® [15]. Minor adverse events will include constipation, diarrhea, nausea, fatigue, dizziness, headache, and alopecia. Serious adverse events will include acute kidney injury, symptomatic electrolyte disturbance, cardiac arrhythmias, symptomatic cholelithiasis, pancreatitis, pyelonephritis, and gout. Preoperative VLED adherence will be calculated as the number of doses of preoperative VLED liquid formulation taken as a proportion of the total doses prescribed as reported by the patient to the best of their abilities.

Secondary outcomes will include: (1) Barriers to preoperative VLED adherence; (2) Thresholds for outcome differences at which patients would be willing to adhere with preoperative VLEDs with liquid formulation; (3) Preoperative weight loss. This will be calculated as the patient’s weight prior to VLED implementation in kilograms subtracted by the patient’s weight immediately preoperatively in kilograms. Height in centimeters will also be calculated and BMI will be computed. We will also compute percent total body weight loss as a measure of weight loss that controls for baseline weight; and (4) Health-related QoL. This will be assessed with an adapted version of the Short-Form 12 (SF-12). This is a truncated version of the SF-36 and has been validated in patients with cardiovascular disease and chronic kidney disease [16,17].

#### Provider survey

The primary outcome will be willingness to prescribe preoperative VLED to patients with obesity undergoing major non-bariatric abdominal surgery for both benign and malignant indications. This will be assessed through five-point Likert scale responses. Secondary outcomes will include: (1) Frequency and type(s) of preoperative weight loss interventions currently being prescribed by practicing surgeons in Canada for patients with obesity undergoing non-bariatric abdominal surgery; (2) Barriers to prescribing preoperative weight loss interventions; (3) Factors associated with prescribing preoperative weight loss; (4) Perceived benefits of prescribing preoperative weight loss; (5) Knowledge surrounding preoperative weight loss options; (6) Perceived difficulty of operating on patients with obesity for major abdominal surgery. These outcomes will be assessed through a combination of five-point Likert scale responses as well as narrative responses provided by surgeons.

### Sample size calculation

#### Patient survey

We propose 35 survey responses to adequately assess our primary outcomes. We justified out proposed sample size based on 95% confidence intervals (CIs) for our safety outcome. Specifically, we hypothesize that 32.5% of patients will experience VLED-associated adverse events based on our previous systematic review and meta-analysis evaluating adult patients with obesity receiving preoperative VLEDs prior to undergoing non-bariatric surgery. Based on review with local surgeons, they would be willing to accept a 50% risk of adverse events for their patients with the use of preoperative VLEDs, as long as the majority of these adverse events were minor (as classified above). Therefore, we calculated the number of patients that would be required to provide sufficiently narrow 95% CIs that would exclude a proportion of 50% assuming that 32.5% of patients reported adverse events. The *cii proportions* function in STATA version 18 was utilized and it was calculated that a sample size of 35 would be required to produce confidence intervals ranging from 16.9% to 49.2%, which we believed was adequate precision. This threshold will be assessed in the present survey to ensure these assumptions are congruent with patient thoughts and expectations.

The cohort of surgeons prescribing preoperative VLEDs for elective non-bariatric intra-abdominal surgery perform between two and six elective intra-abdominal operations per week. Approximately 40% of the patients undergoing intra-abdominal colorectal surgery at our center are living with obesity [7]. Therefore, we expect that one-to-two patients per week will be eligible for inclusion in this survey study. If the survey period lasts for six months, as planned, then we would expect that 24–48 patient survey responses could be collected. Thus, our achieving our planned sample size within our planned study timeline is reasonable.

#### Provider survey

The sample size was calculated using methodology for determining survey sample size with a Likert scale primary outcome published by Park & Jung [18]. In this study, the primary Likert scale outcome that was utilized to calculate the sample size pertained to willingness to prescribe preoperative VLEDs prior to non-bariatric abdominal surgery. The following formula was utilized:

$$n=\frac{z_{\frac{\alpha }{2}}^{2}\cdot C^{2}}{kD^{2}}\{1+(k-1)\rho \},$$

The following are the definitions of the above variables: n, sample size estimate; z, z-score; α, accepted type I error; k, number of items on the Likert scale; D, relative tolerable error in responses; C, coefficient of variation of the population; p, pairwise correlation coefficient. The z-score associated with the accepted type I error is 1.96. The relative tolerable error was set at 5%. The coefficient of variation was set at 0.3 (i.e., the standard deviation of responses will be half the value of the mean) given that respondents tend to avoid extreme responses in Likert scales. The pairwise correlation coefficient was set at 0.3 as the population is relatively heterogenous (i.e., surgeons practicing a variety of different surgical sub-specialties in a variety of different settings across Canada). Given these assumptions and using sample size tables provided by Park & Jung, the required sample size is 60.85, which was rounded to 61 [18].

According to 2020 data from the Canadian Institute for Health Information, there are 2,105 practicing general surgeons in Canada. It is estimated that 115 surgeons in Canada perform exclusively bariatric surgery [19]. If the response rate is estimated conservatively at 10%, this would result in a sample population of 200 surgeons. Therefore, recruitment to a planned sample size of 61 should be feasible within the planned survey timeline of six months.

### Statistical analysis plan

All statistical analyses will be performed on STATA version 18 (StataCorp, College, TX) and Microsoft Excel©. Descriptive statistics will be used to characterize the sample population. Mean and SD will be used for characterizing central tendency and variability for continuous outcomes, respectively. Medians and interquartile ranges (IQR) will be used to characterizing central tendency and variability for ordinal outcomes, respectively. Frequencies (n) and percentages (%) will also be used to characterize the data where appropriate. Likert scale responses will be summarized as medians and IQR, analyzed as ordinal variables, and compared using the Kruskal-Wallis test. Student’s t-tests and chi-squared tests will be used to analyze differences between groups for continuous and dichotomous outcomes, respectively. To determine variables associated with primary outcomes in the patient survey, univariable logistic and linear regression analyses will be performed and presented as odds ratios (OR) with 95% CIs for the following variables: age, sex, BMI, comorbidities, location of residence, type of surgery, and type of disease. To determine variables associated with primary outcomes in the provider survey, univariable logistic and linear regression analyses will be performed and presented as odds ratios (OR) with 95% CIs for the following variables: sex, age, number of years of independent practice, location of practice, type of practice, surgical subspecialty, availability of a bariatric surgery center, availability of a dietician, and availability of a preoperative non-surgical clinic. These multivariable analyses may be adjusted depending on the number of survey responses received. There are no planned interim analyses for the surveys. For the patient survey, *a priori* subgroup analyses will be performed on the basis of type of disease process (i.e., benign, malignant) if sample size allows. For the provider survey, *a priori* subgroup analyses will be performed on the basis of geographic location, type of practice (i.e., academic, non-academic), type of surgery (i.e., upper gastrointestinal surgery, lower gastrointestinal surgery, and other), type of disease process (i.e., benign, malignant), and type of weight loss intervention if sample size allows. Narrative description of survey responses will be provided where applicable.

### Ethical considerations

The patient survey was Approved on August 4^th^, 2023 by the Hamilton Integrated Research Ethics Board (HIREB) (Project #15946). The provider survey was Approved on August 4^th^, 2023 by the HIREB (Project #15946). From a methodological perspective we do not forsee any significant ethical concerns given that we will not be collecting personal health information (PHI). All records will be kept in electronic format under firewall protection (i.e., RedCap®). No personal identifiers will be collected for the purposes of the present surveys. All computers will be stored in a secure location, accessible only by HIREB approved research members. Informed consent for participants will be implied upon survey completion and submission. Once a survey is submitted, it will be unable to be withdrawn. A cover letter at the start of the survey will outline the details of the study and inform participants that by completing the survey they are consenting for collection and analysis of the data therein. This is a minimal risk study, therefore there will be no formal Data Safety Monitoring Board (DSMB).

### Knowledge translation plan

The knowledge translation strategy begins through involvement of the end-users throughout these studies. That is, clinicians who will use the results of this research program for their patients (i.e., colorectal surgeons, general surgeons), will be involved throughout the research process. They have helped develop each of these surveys. Moreover, their opinions will be elucidated with this provider survey and incorporated into our future RCT as well as any subsequent standardized care protocols that result. With regards to the patients who will ultimately be prescribed this preoperative intervention, they will be the focus of the present patient survey in order to elucidate their opinions with regards to preoperative VLEDs and identify barriers to usage. This will hopefully help address barriers and ultimately help improve adherence with this intervention should it become standard of care. Thus, the likelihood that these findings will influence perioperative care for surgical patients with obesity in Canada will be greater because of the end-users who invested in this research from the onset [20]. Once these studies are complete, we will plan to disseminate our findings via peer-reviewed publication, conference presentation(s), and social media. We will aim to stress not only the clinical outcomes of these findings, but the potential outcomes that may result if we intervene on these high-risk patients prior to surgery (i.e., implementation outcomes). Strategies focusing on both clinical and implementation outcomes are most effective in terms of knowledge translation in healthcare [21]. Finally, these surveys will facilitate the completion and dissemination of subsequent studies in this research program (i.e., retrospective study, pilot RCT, definitive RCT) which will contribute to further knowledge generation and translation.

### Protocol amendments

All protocol amendments will be submitted to the Research Ethics Board (REB) as modifications prior to implementation. Amendments will also be communicated during dissemination to both academic and lay audiences.

## Discussion

As surgical patients with obesity becomes increasingly pervasive, developing targeted preoperative optimization protocols for these patients is of growing importance. We feel as though an intensive preoperative weight loss program, such as is afforded by preoperative VLEDs with liquid formulation, can serve as a cornerstone for these protocols. As such, we have developed a research program aimed at assessing the safety and efficacy of preoperative VLEDs with liquid formulation for adult patients with obesity undergoing non-bariatric intra-abdominal surgery. We have previously conducted a systematic review examining prior studies pertaining to the use of preoperative VLEDs in non-bariatric surgery [12]. As a next step in this research program, we aim to elicit both patient and provider opinions with regards to preoperative VLEDs with liquid formulation. Thus, we feel as though these survey studies serve as both clinically and research relevant work. These data will ultimately help inform the design and implementation of an RCT evaluating the efficacy of preoperative VLEDs for patients with obesity undergoing major abdominal surgery.

Given that these are cross-sectional observational studies, the findings will require critical appraisal with a thorough understanding of the potential biases associated with them. Due to the retrospective nature of the present study, recall bias will be a major limitation [22]. Patients who do not experience preoperative weight loss, experience postoperative complications, or sustained a VLED-associated adverse event will likely recall VLED use with negative connotations and their survey responses will likely reflect this. Similarly, providers who have had recent negative experiences with prescribing preoperative weight loss (e.g., VED-associated adverse events, poor patient adherence, recent increase in postoperative morbidity) will likely have survey responses biased by these recent negative experiences. Contrarily, patients who lose substantial weight preoperatively and experience a rapid postoperative recovery, or providers who have had patients do well postoperatively recently, will likely view preoperative weight loss strategies with positive connotations. The survey responses will have to be interpreted with these limitations in mind. In attempt to mitigate this, we will follow recommendations put forth by Khare and Vedel [22]. Secondly, the patient survey will be at significant risk of selection bias. Surgeons may be less willing to prescribe preoperative VLEDs to older, more comorbid patients as it is not a standard of care intervention and thus, we will not capture these higher risk patients in our survey sample. Our findings may not directly apply to this more vulnerable population. Lastly, both surveys will be at risk of sampling bias. We are employing non-probability convenience sampling which is at higher risk than probability sampling in terms of sampling bias. Moreover, we will only have an English written version of the patient survey. This will preclude participation from patients who do not have written English language comprehension. It will also exclude patients who have other comorbidities, impairments, and/or disabilities that prevent the successful completion of the survey. The provider survey will mostly be distributed through large advocacy groups and academic institutions, which may bias towards sampling surgeons practicing in larger centers and in urban areas. Rural surgeons and surgeons less actively involved in advocacy and academic endeavours may not have access to completion of the provider survey. This will limit generalizability of the findings but will improve the feasibility of these studies.

This research program comes at an imperative time for the preoperative care landscape. Preoperative optimization programs, otherwise known as pre-habilitation programs, are appearing with increasing prevalence along with evidence supporting their implementation [23–26]. However, the majority of pre-habilitation data to-date pertain to the old, frail patients undergoing surgery [27,28]. Yet, in the 21^st^ century, the obesity epidemic has started to take center stage [1,29]. In surgery, operating in the abdomen with obesity presents unique challenges for the perioperative team. Patients with obesity undergoing major non-bariatric abdominal surgery have longer operative times, increased intraoperative blood loss, and worse postoperative outcomes, including surgical site infections and venous thromboembolism [5,8,9]. This research program can inform a preoperative optimization pathway for these patients with the aim of combatting these adverse outcomes. These preoperative optimization pathways can subsequently fit within the broader umbrella of Enhanced Recovery After Surgery (ERAS) protocols and may potentially further enhance the patient recovery benefits associated with these well-established protocols.

## Supporting information

S1 File Patient survey. (DOCX)

S2 File Provider survey. (DOCX)
