# Supplementary material for: PREscribing preoperative weight loss prior to major non-bariatric abdominal surgery for patients with Elevated weight: Patient and Provider Survey Protocols (PREPARE surveys)
Source: PLoS One. 2024 Apr 30;19(4):e0302482. doi: 10.1371/journal.pone.0302482 (PMC11060585; doi:10.1371/journal.pone.0302482)
Supplement: S1 File — (DOCX) [file pone.0302482.s001.docx]

**
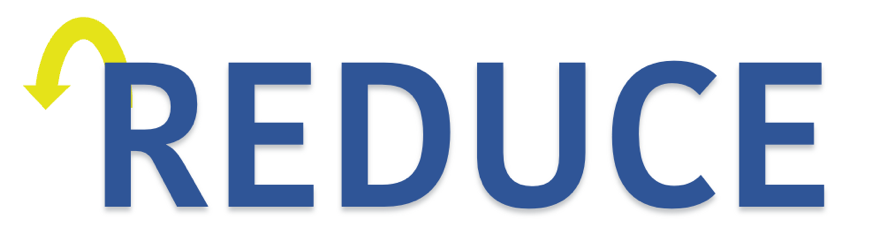
**

Preoperative very low energy diets for obese patients undergoing non-bariatric abdominal surgery: A Patient Perspective Survey

**(REDUCE-PS)**

**Principle Investigator**

Tyler McKechnie MD. McMaster University, Department of Surgery, Division of General Surgery, 1280 Main Street West, Hamilton, Ontario, Canada, L8S 4L8. Email: [tyler.mckechnie@medportal.ca](mailto:tyler.mckechnie@medportal.ca). Phone: (613) 868-9442.

**Supervising Investigators**

Mohit Bhandari MD, PhD, FRCSC. McMaster University, Department of Surgery, Division of Orthopedic Surgery. Hamilton General Hospital, 237 Barton Street East, Hamilon, Ontario, Canada, L8L 2X2. Email: [bhandm@mcmaster.ca](mailto:bhandm@mcmaster.ca). Phone: (905) 541-6057

Cagla Eskicioglu MD, MSc, FRCSC, FASCRS. McMaster University, Department of Surgery, Division of General Surgery. St. Joseph’s Healthcare Hamilton, 50 Charlton Avenue East, Hamilton, Ontario, Canada, L8N 4A6. Email: [eskicio@mcmaster.ca](mailto:eskicio@mcmaster.ca). Phone: (905) 522-1155 ext. 35921. Fax: (905) 540-6515.

**Co-Investigators**

Sameer Parpia PhD. McMaster University, Department of Health Research Methods, Evidence, and Impact. Email: [parpai@mcmaster.ca](mailto:parpai@mcmaster.ca)

Aristithes Doumouras MD, MPH, FRCSC. McMaster University, Department of Surgery, Division of General Surgery. Email: [Aristithes.doumouras@medportal.ca](mailto:Aristithes.doumouras@medportal.ca)

Dennis Hong MD, MSc, FRCSC. McMaster University, Department of Surgery, Division of General Surgery. Email: [dennishong70@gmail.com](mailto:dennishong70@gmail.com)

Nalin Amin MD, FRCSC. McMaster University, Department of Surgery, Division of General Surgery. Email: [amin@mcmaster.ca](mailto:amin@mcmaster.ca)

Edward Passos MD, FRCSC. McMaster University, Department of Surgery, Division of General Surgery. Email: [passosem@mcmaster.ca](mailto:passosem@mcmaster.ca)

Maisa Saddik MSc. McMaster University, Department of Surgery, Division of General Surgery. Email: [saddim@mcmaster.ca](mailto:saddim@mcmaster.ca)

**LETTER OF INFORMATION/CONSENT**

***Purpose of the Study***

You are invited to take part in this research survey aimed at understanding patient experiences with preoperative very low energy diets (i.e., Optifast). We plan on formally studying preoperative very low energy diets with a randomized clinical trial to understand just how effective it can be for patients undergoing non-bariatric surgery. Before doing so, we would like to understand the patient perspective. We have designed this survey to gain an understanding of the patient experience of taking preoperative very low energy diets. Specifically, we would like to understand how compliant patients are with the prescribed regimen, what barriers might exist to being compliant with the regimen, and any perceived risks or benefits that patients may have with regards to this intervention. This research will serve as part of a graduate degree thesis.

***Procedures Involved in the Research***

This is a one-time survey that will take approximately 5-10 minutes to complete. You will be asked to complete either a paper version or electronic version of the survey. We will collect background information on things like your age and sex, information about your surgery, and information about your use of preoperative very low energy diets (i.e., Optifast).

***Potential Harms, Risks, or Discomforts***

It is not likely that there will be any harms or discomforts associated with completion of this survey. The only potential risk associated with participation in the present study is privacy breach. We are collecting minimal personal health information and this information will be stored in a secure location in which only members of the research team can access (i.e., paper forms will be stored in a safe in the research office; electronic forms will be stored on a password protected, encrypted file).

***Potential Benefits***

This research will not benefit you directly. We hope that we will learn more about the lived patient experience with preoperative very low energy diets through your responses. We will then aim to implement this information into the design of our clinical trial, which will hopefully produce results that will be able to improve the care of people around the world undergoing major non-bariatric surgery.

***Confidentiality***

You are participating in this study confidentially. We will not use your name or any information that would allow you to be identified. Your age and other demographic information will be linked to the remainder of your survey responses via a unique participant identification number so that the survey responses are de-identified. These identifiable and de-identifiable data will be separated immediately upon data collection. Quotes left in the comment section may be used in data analysis, but they will be de-identified prior to analysis. The information we collect will either be stored in a safe in a locked office space or on our hospital server under password and fire-wall protection. All computers will be stored in a secure and locked location. Data will be destroyed 20 years after collection.

For the purposes of ensuring proper monitoring of the research study, it is possible that representatives of the HiREB, our institution, and/or regulatory authorities may consult your original research data to check that the information collected for the study is correct and follows proper laws and guidelines. By participating in this study, you authorize such access. By participating in this study, you do not waive any rights to which you may be entitled under the law.

***Participation and Withdrawal***

Your participation in this study is voluntary. You may stop at any point before submission. Choosing not to participate in this chart review will in no way affect your care or treatment.

***Information about the Study Results***

We will share the study results with you via an electronic communication following completion of the survey.

***Questions about the Study***

If you have questions or need more information about the study itself, please contact the principal investigator at: [tyler.mckechnie@medportal.ca](mailto:tyler.mckechnie@medportal.ca)

This study has been reviewed by the Hamilton Integrated Research Ethics Board (HiREB). The HiREB is responsible for ensuring that participants are informed of the risks associated with the research, and that participants are free to decide if participation is right for them. If you have any questions about your rights as a research participant, please call the Office of the Chair, HiREB, at 905.521.2100 x 42013.

**CONSENT**

***By proceeding to complete the survey, you are providing implied consent for your participation in the present study.***

**SURVEY INSTRUCTIONS**

1. Please complete the following survey if you are a patient who underwent major non-bariatric abdominal surgery at St. Joseph’s Healthcare Hamilton and were prescribed 2-to-3 weeks of Optifast® 900 prior to undergoing surgery.
2. All responses will be anonymized and kept confidential.
3. By signing the informed consent form above and completing this survey, consent for the use of the data for research purpose is assumed.
4. Please note, your participation in this survey is voluntary and you may stop at any point before submission. The decision to participate and your responses will have no bearing on the clinical care you receive.
5. Co-enrollment in another study is allowed while participating in this study.
6. This study has been reviewed by the Hamilton Integrated Research Ethics Board (HiREB). The HiREB is responsible for ensuring that participants are informed of the risks associated with the research, and that participants are free to decide if participation is right for them. If you have any questions about your rights as a research participant, please call the Office of the Chair, HiREB, at 905.521.2100 x 42013
7. De-identified quotes may be used from the comments section in the final research report.
8. All data from this survey will be securely stored on a password and firewall protected computer in a locked office with restricted access.
9. If we have not captured some of your thoughts, please include pertinent information in the comments section at the end of the survey.
10. This survey will take approximately 5-10 minutes to complete. Please submit the completed survey once you have filled all sections and are satisfied with your answer.

| Section A: Demographic Information |
| --- |

1. **Date:** ________________________________
2. **Age:** ________________________________
3. **Sex:** Male Female Other Prefer not to Answer
4. **Where do you live (city/town and province)?**

____________________________________________________________________

| Section B: Treatment Information |
| --- |

1. **Date of surgery:** ________________________________
2. **Did you receive any chemotherapy or radiation before your surgery?**

Yes No

1. **Did you receive any chemotherapy after your surgery?**

Yes No

| Section C: Prescribed Preoperative Weight Loss |
| --- |

1. **Were you prescribed Optifast® 900 (i.e., weight loss drinks) before your surgery**?

Yes No

1. **How long were you prescribed weight loss drinks for before surgery?**

1 week 2 weeks 3 weeks

Other: _______________________

1. **How much of the prescribed doses of weight loss drinks did you take?**

0% 10% 20% 30% 40% 50% 60% 70% 80% 90% 100%

1. **If you did not take all of the prescribed doses of weight loss drinks, why not?**

Hunger Taste Cost

Difficulty getting the medications Complications Didn’t want to

Not seeing change Other: ______________________________

1. **Is there anything that would have made it easier to take all of the prescribed doses of weight loss drinks?**

Less costly Better taste More food options

More frequent drinks Less frequent drinks Easier to get them

Obvious change/weight loss Other: __________________________________

1. **How much weight did you lose from the time of starting the weight loss drinks to immediately prior to surgery?**

__________________________________________________________________lbs

1. **How much weight did you lose from the time of starting the weight loss drinks to now?**

__________________________________________________________________lbs

1. **Did you experience any of the following minor complications when taking the weight loss drinks ?**

Constipation Diarrhea Nausea

Fatigue Dizziness Headache

Hair loss Other: ____________________________________

1. **Did you experience any of the following major complications when taking the weight loss drinks?**

Admission to hospital Severe Dehydration Abnormal Heart Rhythm

Electrolyte imbalance Gallbladder stones Kidney Stones

Gout Pancreatitis Other: __________________

1. **If you knew that weight loss drinks made surgery easier for your surgeon, would your willingness to take all of the doses increase?**

1 2 3 4 5

Not at all Somewhat Absolutely yes

1. **If you knew that weight loss drinks decreased the risk of experiencing complications after surgery, would your willingness to take all of the doses increase?**

1 2 3 4 5

Not at all Somewhat Absolutely yes

1. **If you knew that weight loss drinks decreased the amount of time you spent in hospital after surgery, would your willingness to take all of the doses increase?**

1 2 3 4 5

Not at all Somewhat Absolutely yes

1. **If you knew that weight loss drinks improved your quality of life, would your willingness to take all of the doses increase?**

1 2 3 4 5

Not at all Somewhat Absolutely yes

1. **If the duration of preoperative weight loss drinks was two weeks instead of three weeks, would your willingness to take all of the doses increase?**

1 2 3 4 5

Not at all Somewhat Absolutely yes

1. **Are you planning on continuing with weight loss strategies after surgery?**

1 2 3 4 5

Not at all Somewhat Absolutely yes

| Section D: Quality of Life |
| --- |

**************Adapted from the SF-12 Health Related Quality of Life Questionnaire**********

****Attempt to answer from the perspective of your health immediately prior to surgery, while taking the preoperative weight loss product****

1. **In general, would you say your health is:**

Excellent Very Good Good Fair Poor

1. **Does your health now limit you in the following activities?**
   1. **Moderate activities, such as moving a table, pushing a vacuum cleaner, bowling, or playing golf.**

Yes – limited a lot Yes – limited a little No

- 1. **Climbing several flights of stairs.**

Yes – limited a lot Yes – limited a little No

1. **During the past 4 weeks, have you had any of the following problems with your work or other daily activities as a result of your physical health?**
   1. **Accomplished less than you would like**

Yes No

- 1. **Were limited in the kind of work or other activities**

Yes No

1. **During the past 4 weeks, have you had any of the following problems with your work or other regular daily activities as a result of any emotional problems (such as feeling depressed or anxious)?**
   1. **Accomplished less than you would like**

Yes No

- 1. **Were limited in the kind of work or other activities**

Yes No

1. **During the past 4 weeks, how much did pain interfere with your normal work (including work outside the home and housework)?**

Not at all A little bit Moderately Quite a bit Extremely

1. **How much of the time during the past 4 weeks have you felt calm and peaceful?**

All of the time Most of the time A good bit of the time

Some of the time A little of the time None

1. **How much of the time during the past 4 weeks did you have a lot energy?**

All of the time Most of the time A good bit of the time

Some of the time A little of the time None

1. **How much of the time during the past 4 weeks have you felt down-hearted and blue?**

All of the time Most of the time A good bit of the time

Some of the time A little of the time None

1. **During the past 4 weeks, how much time has your physical or emotional problems interfered with your social activities (like visiting friends, relatives, etc.)?**

All of the time Most of the time A good bit of the time A little of the time None

| Section E: Comments |
| --- |

Thank you for participating in this survey! Your input is appreciated. For any questions about this survey or the associated research project, please contact Dr. Tyler McKechnie ([tyler.mckechnie@medportal.ca](mailto:tyler.mckechnie@medportal.ca)).
