# Supplementary material for: PREscribing preoperative weight loss prior to major non-bariatric abdominal surgery for patients with Elevated weight: Patient and Provider Survey Protocols (PREPARE surveys)
Source: PLoS One. 2024 Apr 30;19(4):e0302482. doi: 10.1371/journal.pone.0302482 (PMC11060585; doi:10.1371/journal.pone.0302482)
Supplement: S2 File — (DOCX) [file pone.0302482.s002.docx]

Prescribing Preoperative Weight Loss Prior to Major Non-Bariatric Abdominal Surgery in Obese Patients: A National Survey

**Principal Investigator**

Tyler McKechnie MD. McMaster University, Department of Surgery, Division of General Surgery, 1280 Main Street West, Hamilton, Ontario, Canada, L8S 4L8. Email: [tyler.mckechnie@medportal.ca](mailto:tyler.mckechnie@medportal.ca). Phone: (613) 868-9442.

**Supervising Investigators**

Mohit Bhandari MD, PhD, FRCSC. McMaster University, Department of Surgery, Division of Orthopedic Surgery. Hamilton General Hospital, 237 Barton Street East, Hamilon, Ontario, Canada, L8L 2X2. Email: [bhandm@mcmaster.ca](mailto:bhandm@mcmaster.ca). Phone: (905) 541-6057

Cagla Eskicioglu MD, MSc, FRCSC, FASCRS. McMaster University, Department of Surgery, Division of General Surgery. St. Joseph’s Healthcare Hamilton, 50 Charlton Avenue East, Hamilton, Ontario, Canada, L8N 4A6. Email: [eskicio@mcmaster.ca](mailto:eskicio@mcmaster.ca). Phone: (905) 522-1155 ext. 35921. Fax: (905) 540-6515.

**Co-Investigators**

Sameer Parpia PhD. McMaster University, Department of Health Research Methods, Evidence, and Impact. Email: [parpai@mcmaster.ca](mailto:parpai@mcmaster.ca)

Aristithes Doumouras MD, MPH, FRCSC. McMaster University, Department of Surgery, Division of General Surgery. Email: [Aristithes.doumouras@medportal.ca](mailto:Aristithes.doumouras@medportal.ca)

Yung Lee MD. McMaster University, Department of Surgery, Division of General Surgery. Email: [yung.lee@mcmaster.ca](mailto:yung.lee@mcmaster.ca)

Maisa Saddik MSc. McMaster University, Department of Surgery, Division of General Surgery. Email: [saddim@mcmaster.ca](mailto:saddim@mcmaster.ca)

**Survey Instructions**

1. Please complete the following survey if you are currently a board-certified, practicing surgeon in Canada who performs elective non-bariatric intra-abdominal surgery.
2. All responses will be anonymized and kept confidential.
3. By completing this survey, implied consent for the use of the data for research purpose is assumed.
4. Please note, your participation in this survey is voluntary and you may stop at any point before submission. Once responses are submitted, they cannot be withdrawn.
5. Co-enrollment in another study is allowed while participating in this study.
6. This study has been reviewed by the Hamilton Integrated Research Ethics Board (HiREB). The HiREB is responsible for ensuring that participants are informed of the risks associated with the research, and that participants are free to decide if participation is right for them. If you have any questions about your rights as a research participant, please call the Office of the Chair, HiREB, at 905.521.2100 x 42013.
7. If we have not captured some of your preferences, please include pertinent information in the comments section at the end of the survey.
8. If there are any important aspects of your current preoperative weight loss prescribing practice that we did not capture this survey, please include pertinent information in the comments section at the end of the survey.
9. This survey will take approximately 10 minutes to complete.

**Survey Details**

The obese surgical patient is becoming increasingly pervasive. For major abdominal surgery this presents a significant challenge as operating in a field with significant subcutaneous and visceral adiposity is difficult regardless of operative approach. For this reason, patients undergoing bariatric surgery are prescribed very low energy diets (VLEDs) for several weeks prior to their operation. However, VLEDs are much less commonly used in non-bariatric abdominal surgery.

We conducted a systematic review aimed at identifying studies that evaluated VLEDs in non-bariatric surgery. Thirteen studies were found, nine of which evaluated obese patients undergoing abdominal surgery. While the evidence was heterogenous, the available data suggests preoperative VLEDs are safe, well tolerated, and result in significant preoperative weight loss for these patients. As such, we are in the process of designing a randomized controlled trial (RCT) aimed at assessing the efficacy of VLEDs at reducing operative difficulty and improving postoperative outcomes.

Prior to proceeding with this RCT, we are interested in assessing the willingness to prescribe preoperative VLEDs and other weight loss strategies. As such, we have designed this cross-sectional national survey for practicing surgeons in Canada to gain an understanding of the feasibility of this intervention as well as the potential impact it may have on surgical practices across the Country.

| Section A: Demographic Information |
| --- |

1. Age: (write in)
2. How many years have you been practicing as an independent surgeon? (write in)
3. Sex (drop down menu)
4. In which city do you practice? (write in)
5. In which province/territory do you primarily practice? (drop down menu)
6. Which surgical specialty do you primarily practice? (drop down menu)
7. Do you perform oncology cases? (drop down menu)
8. What intraoperative and/or postoperative outcomes are most important to you? (drop down menu)

| Section B: Institution Information |
| --- |

1. How would you classify your current position? (drop down menu)
2. How many other surgeons do you currently practice with at your institution? (write in)
3. Is there bariatric surgery available at your center? (drop down menu)
4. Do you have a dietician service available at your center? (drop down menu)
5. Do you have a preoperative clinic that you send patients to be seen prior to major abdominal surgery? (drop down menu)

| Section C: Prescribing Preoperative Weight Loss |
| --- |

1. Do you discuss preoperative weight loss with obese patients undergoing major abdominal surgery? (Five Point Likert Scale)
2. What is your “BMI cut-off” at which you begin recommending preoperative weight loss? (drop down menu)
3. What preoperative weight loss strategies do you recommend? (drop down menu)
4. If you prescribe liquid formula supplementation for very low energy diets, which formulation do you prescribe? (drop down menu)
5. If you prescribe liquid formula supplementation for very low energy diets, how long do you prescribe them for? (drop down menu)
6. How willing are you to prescribe preoperative very low energy diets with liquid formula supplementation to obese patients undergoing major abdominal surgery? (five point Likert Scale)
7. Are there any institutional/regional barriers to prescribing very low energy diets with liquid formula supplementation to obese patients undergoing major abdominal surgery? (drop down menu)
8. How much do you think obesity impacts your operations? (five point Likert scale)
9. How much do you think preoperative weight loss would impact the technical ease of your operations? (five point Likert scale)
10. How much do you think preoperative weight loss would impact the postoperative recovery of your patients? (five point Likert scale)
11. What differences in intraoperative and/or postoperative outcomes would you want to see to convince you to start prescribing preoperative weight loss with liquid formulation (e.g., Optifast)? (drop down menu)
12. Are there particular surgeries in which you believe prescribing preoperative weight loss would be most beneficial? (write in)
13. Are there particular surgeries in which you believe prescribing preoperative weight loss would be least beneficial? (write in)
14. How would you rate your knowledge on preoperative weight loss options? (five point Likert scale)
15. How would you rate your knowledge on very low energy diets with liquid formulation (e.g., Optifast) specifically? (five point Likert scale)
16. How many kilocalories do you think a very low energy diet should target daily? (write in)

| Section D: Oncology Patients |
| --- |

1. Do you become apprehensive about prescribing preoperative weight loss interventions to obese patients undergoing surgery for cancer? (five point Likert Scale)
2. How willing would you be to prescribe preoperative very low energy diets with liquid formula supplementation to obese patients undergoing surgery for cancer? (five point Likert Scale)

| Section E: Comments |
| --- |

(Text box)

Thank you for participating in this survey! Your input is appreciated. For any questions about this survey or the associated research project, please contact Dr. Tyler McKechnie ([tyler.mckechnie@medportal.ca](mailto:tyler.mckechnie@medportal.ca)).
